# Association between Peptic Ulcer Disease and Periodontitis: A Nationwide Population-Based Case-Control Study in Taiwan

**DOI:** 10.3390/ijerph15050912

**Published:** 2018-05-04

**Authors:** Hui-Chieh Yu, Tsung-Po Chen, Chia-Yi Wei, Yu-Chao Chang

**Affiliations:** 1 School of Dentistry, Chung Shan Medical University, Taichung 40201, Taiwan; yujessica7@gmail.com (H.-C.Y.); feedfly210@hotmail.com (C.-Y.W.); 2 Department of Family Medicine, China Medical University Hospital, Taichung 40402, Taiwan; tsungpo88@gmail.com; 3 Department of Dentistry, Chung Shan Medical University Hospital, Taichung 40201, Taiwan

**Keywords:** periodontitis, peptic ulcer disease, gastric ulcer, duodenal ulcer, *Helicobacter pylori*

## Abstract

Previous studies have suggested that peptic ulcer disease (PUD) including stomach and duodenal ulcers might be associated with periodontitis (PD); however, no clear conclusions have been reached thus far. In this retrospective case-control study, we aimed to investigate the association between PUD and PD by using a large population-based dataset in Taiwan. A population-based retrospective case control study was conducted using the Longitudinal Health Insurance Database 2010 (LHID2010) derived from the National Health Insurance Research database (NHIRD) in Taiwan from 2000 to 2013. The case and control group were matched with gender, age, urbanization level, socioeconomic status, and Charlson comorbidity index (CCI) by using the propensity score method at a 1:1 ratio. A total of 177,240 cases and 177,240 control patients were included in this study, with an average age of 46.96 ± 11.76 years. The risk of PUD for patients diagnosed with PD was 1.15-fold when compared with those without PD (OR, 1.15; 95% CI, 1.12–1.18). This population-based case control study demonstrated a significantly positive association between PUD and PD in Taiwan.

## 1. Introduction

Peptic ulcer disease (PUD) is a disease located in the gastric or duodenal part of the gastrointestinal tract that mainly involves the mucosa layer. The two main risk factors for PUD are *Helicobactor pylori* (*H. pylori*) infection and medication consumption, especially of nonsteroid anti-inflammatory drugs (NSAIDs) [1]. The prevalence of PUD ranges from 0.12 to 1.5% and increases with age [2]. The major symptom of uncomplicated PUD is upper abdominal dyspepsia such as bloating, early satiety, and nausea [3].

Periodontitis (PD) is defined as an inflammatory disease of the supporting tissues surrounding the teeth [4]. Inflammatory periodontal disease results from a complex interaction between the subgingival biofilm and the host immune–inflammatory events [5]. In response to the challenge presented by the bacteria, progressive destruction in the gingival and periodontal tissues occurs. A four-year national survey in the U.S. showed that 46% of adults aged ≥30 years had PD, with 8.9% having severe PD. Asian Americans experienced a mean attachment loss of 1.95 mm and 15.4% had clinical attachment loss (CAL) ≥7 mm [6]. In addition, a study of PD prevalence in Taiwan showed that the prevalence of PD gradually rose from 11.5% in 1997 to 19.59% in 2013 during the 17-year study period [7].

*H. pylori* infection plays a crucial role in the pathogenesis of PUD. *H. pylori* infection is involved in various gastroduodenal pathologies, and evokes the production of proinflammatory interleukin-1beta [8], leading to the reduction of blood flow to the gastroduodenal tract and increasing the risk of peptic ulcers [9]. *H. pylori* can colonize not only in the stomach, but also in the oral cavity. The oral cavity may be a reservoir for *H. pylori* and a potential source for infection of the stomach [10]. Previous studies have provided evidence that PUD might be associated with PD [11,12,13,14]; however, no clear conclusions have been reached thus far. Therefore, we conducted a population-based case-control study to investigate the putative association from the database of Taiwanese National Health Insurance Research (NHIRD).

## 2. Materials and Methods

### 2.1. Data Source and Study Design

The dataset used in this case-control study was derived from the NHIRD in Taiwan. The Longitudinal Health Insurance Database 2010 (LHID2010) was created and released to the public by the National Health Research Institute (NHRI) and includes all the original claims data and registration files from 2000 to 2013 for one million individuals randomly sampled from the Registry for Beneficiaries of the National Health Institute (NHI) program in 2010 in Taiwan. There are approximately 27.38 million individuals on this registry, with a coverage rate of more than 99%. The disease diagnoses were defined according to the International Classification of Diseases, Ninth Revision, Clinical Modification (ICD-9-CM). This study was approved by the Chung Shan Medical University Hospital Ethics Review Board (CSMUH No.CS2-15017, Date: 10/04/2015).

### 2.2. Selection of Case and Control 

We used the ICD-9-CM diagnosis codes to identify the subjects with PUD. Gastric ulcers (ICD-9-CM code 531), duodenal ulcers (ICD-9-CM codes 532), and peptic ulcers (ICD-9-CM codes 533) occurring between January 2000 and December 2013 were retrieved from the database. Patients aged under 18 years old, aged older than 65 years old, who had withdrawn from the program, or for whom data were missing were excluded from this study. Patients who had a past history of stomach surgery, gastric cancer, or Zollinger–Ellison syndrome were also excluded. Cases were selected under a minimum of three diagnoses of PUD (ICD-9-CM codes: 531, 532, 533) after outpatient department visits. For each case, we conditionally selected comparison subjects from the general population with matched gender, age, urbanization level, socioeconomic status, and Charlson comorbidity index (CCI) [15] by using a propensity score method at a 1:1 ratio. Urbanization was categorized into three levels—urban, suburban, and rural areas—based on the classification scheme proposed by Liu et al. [16]. Socioeconomic status was defined by monthly income (<NT$ 20,000, NT$ 20,000–40,000, and >NT$ 40,000). The Charlson Comorbidity Index (CCI) was used to estimate the overall systemic health. Each increment in the CCI score indicates a stepwise increase in cumulative mortality.

### 2.3. Exposure Assessment

**Figure 1:**
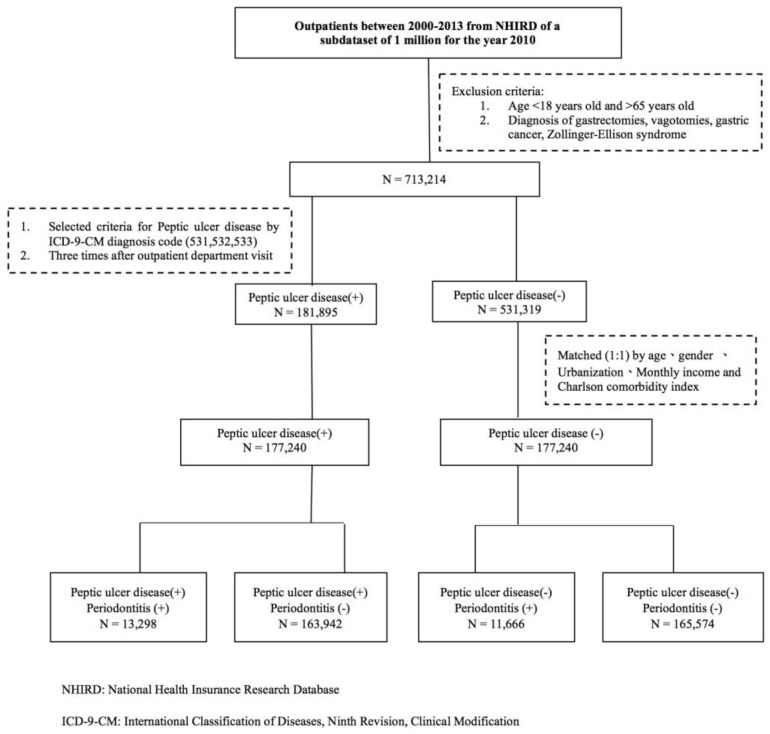
The flow diagram of the study participants.

To validate the PD diagnosis sourced from the administrative database, we identified PD cases not just by the ICD-9-CM diagnosis codes (ICD-9-CM codes 523.3, 523.4, and 523.5), but also by the treatment codes (91006C, 91007C, 91008C 91009B, and 91010B) of the NHI system. The periodontal treatment code including subgingival curettage and periodontal flap operation is described in Table 1. The NHI system allows dental clinicians to perform periodontal treatment only if beneficiaries meet the criteria as prescribed. Potential confounding factors including sex, age, socioeconomic factors, urbanization, and CCI were identified and categorized. The flowchart of the study is shown in Figure 1.

### 2.4. Statistical Analysis

Statistical analyses were performed using Student’s *t*-test for continuous variables and the chi-squared test for categorical variables. The odds ratio (OR) between the case and control groups was analyzed with the chi-square test. A multinomial logistic regression model was used for subgroup analysis. All results are presented in ORs and 95% confidence intervals (CIs). A two-sided *p* < 0.05 was considered statistically significant. All analyses were conducted in SPSS version 22 (SPSS Inc., Chicago, IL, USA).

## 3. Results

As shown in Table 2, 177,240 cases and 177,240 control patients were included in this study, with an average age of 46.96 ± 11.76 years. In the case group, 81,965 (46.25%) cases were male and 95,275 (53.75%) were female. Characteristics including age, gender, urbanization, monthly income, and CCI score were not significantly different between the 1:1 matched cases and the control group (*p* > 0.05).

The risk of PUD for patients diagnosed with PD was 1.15-fold when compared with those without PD (OR, 1.15; 95% CI, 1.12–1.18) in Table 3.

## 4. Discussion

In this case-control study, we revealed that PD was positively associated with PUD (OR, 1.15; 95% CI, 1.12–1.18). A significantly higher proportion with a diagnosis of PD was found among patients of PUD when compared with the control group.

PD is derived from a complex interaction between the subgingival biofilm and the host immune–inflammatory events. The net result of these inflammatory changes could break down the fibers of the periodontal ligament, leading to clinical loss of attachment, resorption of the alveolar bone with increased probing depth formation, and tooth loss [17]. Moreover, the chronic inflammation is associated with multiple systemic diseases including diabetes mellitus [18], rheumatoid arthritis [19], and cardiovascular disease [20]. In recent scientific research, few studies [11,12,13,14,21] have proposed the positive association between PD and PUD. Our investigation showed similar results. However, those studies recruited fewer participants and some studies [11,14] used self-administered questionnaires for disease evaluation or were confined to a specific group like health professionals [11], which made the outcomes limited for interpretation.

This case-control study had more strength than the previous cross-sectional design of studies. To the best of our knowledge, this is the first large-scale population-based study to investigate the association between PD and PUD. With large numbers of participants derived from the NHIRD, the longitudinal sampling dataset from 2000 to 2013 made this study more representative. The selection of cases was on the basis of ICD-9 diagnostic codes plus the diagnoses of at least three outpatient department visits to improve the validity of our measurements. We also solidified the diagnosis of PD in corresponding periodontal treatment procedure. A well-matched case and control group can minimize the potential biases of gender, age, urbanization, socioeconomic status, and CCI.

Despite considerable debate surrounding the pathophysiology underlying the association between PD and PUD, the influence of PD on PUD could be explained in several ways. Two important risk factors for PUD are *H. pylori* infection and NSAID use. *H. pylori* is a Gram-negative bacterium that colonizes mainly in the gastric mucosa and its presence has been universally associated with chronic gastritis, peptic ulcers, and mucosa-associated lymphoid tissue lymphoma [3,22]. The maintenance of gastrointestinal tract hormones plays a fundamental role in the regulation of body physiological activity; however, *H. pylori* infection might cause an imbalance of hormones [23,24,25]. The prevalence of *H. pylori* infection is high and is more than 50% in most countries worldwide [26]. Many studies have mentioned that the periodontal reservoir harvested *H. pylori* and caused inflammation, which induced PD [27,28,29]. Some studies have shown that the presence of *H. pylori* in dental plaque via different detection methods might be a possible source of infection and reinfection of the stomach [21,30,31,32]. In summary, these works have shown the positive association between *H. pylori* and periodontal pathogens. Other studies have also discussed the effects of periodontal therapy on oral and gastric *H. pylori* [30,33,34]. Those who received both anti-*H. pylori* and periodontal therapy had higher eradication rates when compared with those who only received anti-*H. pylori* therapy [30,33]. In addition, a meta-analysis suggested that periodontal therapy could improve the outcome of eradication therapy of PUD [34].

Due to our strict assessment of PD from NHIRD by using both ICD-9 diagnostic codes and treatment codes, patients with a diagnosis of PD corresponded to a proper periodontal treatment. It must be emphasized that the strict assessment of exposure caused a low prevalence rate of PD in this study. Although it did not reach a statistically significant difference between the surgical and nonsurgical periodontal treatment procedures in the stratified analysis, it seemed that the ORs of PUD were attenuated with the intensity of treatment. Periodontal treatment that includes surgical and nonsurgical periodontal therapy along with patient education for plaque control could eradicate the oral *H. pylori*. The reduction in microbial etiological factors after periodontal treatment might lead to a reduced risk of PUD. Recently, a review of periodontium and *H. pylori* supported the view that periodontal treatment may play an effective role in the management of *H. pylori*-associated gastric disease [35]. Further analyses to clarify the impact of the intensity of periodontal therapy still need to be carried out.

It has been reported that both PD and PUD are associated with low socioeconomic status [36,37,38,39]. In our study, approximately 78% of patients were considered to be socioeconomically disadvantaged due to a low monthly income (<NT$ 20,000). In spite of increasing trends of poorer health among those with a lower socioeconomic status, there are still some risk factors that are unevenly distributed across socioeconomic status, such as smoking habits [40]. One major limitation of our study retrieved from the NHIRD was the lack of anthropometric data and health-related behaviors or status such as betel nut chewing habit, cigarette smoking, and alcohol consumption. This limitation is caused by the inherent shortcomings of the administrative database. Nevertheless, the use of a nationwide population-based database can provide sufficient sample size, generalizability, and statistical power to assess the association of diseases. It may be beneficial to provide additional data analysis and assist in planning treatment strategies.

## 5. Conclusions

In conclusion, this study demonstrated a positive association between PUD and PD. The results showed the clinical value for both dental and gastroenterological practitioners, suggesting a newer treatment consideration for PUD. However, further prospective studies are needed to confirm the causal relationship between these two diseases.

## Figures and Tables

**Table 1:** Periodontal treatment code of the NHI system.

Coding	Periodontitis Treatment	Description
Nonsurgical		
91006C	Subgingival curettage (Root planing)—full mouth	* At least 1 pocket depth ≥5 mm of 6 probing sites in one tooth. * Records of pocket depth and X-ray prior to treatment are needed. * Procedure is allowed in dental clinics, local community hospitals, metropolitan hospitals, and medical centers.
91007C	Subgingival curettage (Root planing)—1/2 arch	
91008C	Subgingival curettage (Root planing)—localized, less than 3 teeth	
Surgical		
91009B	Periodontal flap operation—localized, less than 3 teeth	*At least 1 pocket depth ≥5 mm of 6 probing sites in one tooth. * Records of pocket depth and x-ray prior to treatment are needed. * Procedure is allowed in local community hospitals, metropolitan hospitals, and medical centers.
91010B	Periodontal flap operation—1/3 arch, 4 to 6 teeth	

**Table 2:** Comparisons in demographic characteristics in patients with PUD.

Variable	Total (*n* = 354,480)		PUD (*n* = 177,240)		Non-PUD (*n* = 177,240)		*p*-Value	
	Population	%	Population	%	Population	%		
Periodontitis	24,964	7.04	13,298	7.5	11,666	6.58		
Age (years)	46.96 ± 11.76		46.95 ± 11.75		46.96 ± 11.76		0.83	
Age groups (years)							0.72	
18–25	15,772	4.45	7885	4.45	7887	4.45		
26–35	53,348	15.05	26,670	15.05	26,678	15.05		
36–45	83,196	23.47	41,565	23.45	41,631	23.49		
46–55	98,874	27.89	49,615	27.99	49,259	27.79		
56–65	103,290	29.14	51,505	29.06	51,785	29.22		
Gender							0.91	
Female	190,585	53.76	95,275	53.75	95,310	53.77		
Male	163,895	46.24	81,965	46.25	81,930	46.23		
Urbanization							0.34	
Urban	212,059	59.82	105,886	59.74	106,173	59.9		
Suburban	116,103	32.75	58,088	32.77	58,015	32.73		
Rural	26,318	7.42	13,266	7.48	13,052	7.36		
Monthly income							0.89	
<NT$ 20,000	275,262	77.65	137,588	77.63	137,674	77.68		
NT$ 20,000~40,000	53,037	14.96	26,526	14.97	26,511	14.96		
>NT$ 40,000	26,181	7.39	13,126	7.41	13,055	7.37		
CCI							0.11	
0	194,648	54.91	97,318	54.91	97,330	54.91		
1	79,339	22.38	39,464	22.27	39,875	22.5		
≧2	80,493	22.71	40,458	22.83	40,035	22.59		

PUD: Peptic ulcer disease; CCI: Charlson comorbidity index.

**Table 3:** Odds ratio for peptic ulcer disease in those with a diagnosis of periodontitis.

	PUD	Non-PUD	OR
Periodontitis			
No	163,942 (92.5%)	165,574 (93.4%)	1
Yes	13,298 (7.5%)	11,666 (6.6%)	1.15 (1.12–1.18)

PUD: peptic ulcer disease; OR: odds ratio.

## References

[1] Malfertheiner P. (2011). The intriguing relationship of helicobacter pylori infection and acid secretion in peptic ulcer disease and gastric cancer. Dig. Dis..

[2] Sung J.J., Kuipers E.J., El-Serag H.B. (2009). Systematic review: The global incidence and prevalence of peptic ulcer disease. Aliment. Pharmacol. Ther..

[3] Suerbaum S., Michetti P. (2002). Helicobacter pylori infection. N. Engl. J. Med..

[4] Williams R.C. (1990). Periodontal disease. N. Engl. J. Med..

[5] Laudenbach J.M., Simon Z. (2014). Common dental and periodontal diseases: Evaluation and management. Med. Clin. N. Am..

[6] Eke P.I., Dye B.A., Wei L., Slade G.D., Thornton-Evans G.O., Borgnakke W.S., Taylor G.W., Page R.C., Beck J.D., Genco R.J. (2015). Update on prevalence of periodontitis in adults in the United States: Nhanes 2009 to 2012. J. Periodontol..

[7] Yu H.C., Su N.Y., Huang J.Y., Lee S.S., Chang Y.C. (2017). Trends in the prevalence of periodontitis in Taiwan from 1997 to 2013: A nationwide population-based retrospective study. Medicine (Baltimore).

[8] Warzecha Z., Dembinski A., Ceranowicz P., Dembinski M., Sendur R., Pawlik W.W., Konturek S.J. (2002). Deleterious effect of Helicobacter pylori infection on the course of acute pancreatitis in rats. Pancreatology.

[9] Leung F.W., Su K.C., Pique J.M., Thiefin G., Passaro E., Guth P.H. (1992). Superior mesenteric artery is more important than inferior mesenteric artery in maintaining colonic mucosal perfusion and integrity in rats. Dig. Dis. Sci..

[10] Jia C.L., Jiang G.S., Li C.H., Li C.R. (2009). Effect of dental plaque control on infection of helicobacter pylori in gastric mucosa. J. Periodontol..

[11] Boylan M.R., Khalili H., Huang E.S., Michaud D.S., Izard J., Joshipura K.J., Chan A.T. (2014). A prospective study of periodontal disease and risk of gastric and duodenal ulcer in male health professionals. Clin. Transl. Gastroenterol..

[12] Kaneto C., Toyokawa S., Inoue K., Inoue M., Senba T., Suyama Y., Miyoshi Y., Kobayashi Y. (2012). Association between periodontal disease and peptic ulcers among Japanese workers: My health up study. Glob. J. Health Sci..

[13] Namiot D.B., Namiot Z., Kemona A., Golebiewska M. (2006). Peptic ulcers and oral health status. Adv. Med. Sci..

[14] Khader Y.S., Rice J.C., Lefante J.J. (2003). Factors associated with periodontal diseases in a dental teaching clinic population in northern Jordan. J. Periodontol..

[15] Charlson M.E., Pompei P., Ales K.L., MacKenzie C.R. (1987). A new method of classifying prognostic comorbidity in longitudinal studies: Development and validation. J. Chronic Dis..

[16] Liu C.Y., Hung Y.T., Chuang Y.L., Chen Y.J., Weng W.S., Liu J.S., Lia K.Y. (2006). Incorporating development stratification of Taiwan townships into sampling design of large scale health interview survey. J. Health Manag..

[17] Kim J., Amar S. (2006). Periodontal disease and systemic conditions: A bidirectional relationship. Odontology.

[18] Preshaw P.M., Alba A.L., Herrera D., Jepsen S., Konstantinidis A., Makrilakis K., Taylor R. (2012). Periodontitis and diabetes: A two-way relationship. Diabetologia.

[19] Koziel J., Mydel P., Potempa J. (2014). The link between periodontal disease and rheumatoid arthritis: An updated review. Curr. Rheumatol. Rep..

[20] Blaizot A., Vergnes J.N., Nuwwareh S., Amar J., Sixou M. (2009). Periodontal diseases and cardiovascular events: Meta-analysis of observational studies. Int. Dent. J..

[21] Namiot D.B., Leszczynska K., Namiot Z., Chilewicz M., Bucki R., Kemona A. (2010). The occurrence of helicobacter pylori antigens in dental plaque; an association with oral health status and oral hygiene practices. Adv. Med. Sci..

[22] McColl K.E. (2010). Clinical practice. Helicobacter pylori infection. N. Engl. J. Med..

[23] Ceranowicz P., Warzecha Z., Dembinski A. (2015). Peptidyl hormones of endocrine cells origin in the gut—Their discovery and physiological relevance. J. Physiol. Pharmacol..

[24] Cieszkowski J., Warzecha Z., Ceranowicz P., Ceranowicz D., Kusnierz-Cabala B., Pedziwiatr M., Dembinski M., Ambrozy T., Kaczmarzyk T., Pihut M. (2017). Therapeutic effect of exogenous ghrelin in the healing of gingival ulcers is mediated by the release of endogenous growth hormone and insulin-like growth factor-1. J. Physiol. Pharmacol..

[25] Warzecha Z., Kownacki P., Ceranowicz P., Dembinski M., Cieszkowski J., Dembinski A. (2013). Ghrelin accelerates the healing of oral ulcers in non-sialoadenectomized and sialoadenectomized rats. J. Physiol. Pharmacol..

[26] Eusebi L.H., Zagari R.M., Bazzoli F. (2014). Epidemiology of helicobacter pylori infection. Helicobacter.

[27] Payao S.L., Rasmussen L.T. (2016). Helicobacter pylori and its reservoirs: A correlation with the gastric infection. World J. Gastrointest. Pharmacol. Ther..

[28] Hu Z., Zhang Y., Li Z., Yu Y., Kang W., Han Y., Geng X., Ge S., Sun Y. (2016). Effect of helicobacter pylori infection on chronic periodontitis by the change of microecology and inflammation. Oncotarget.

[29] Anand P.S., Nandakumar K., Shenoy K.T. (2006). Are dental plaque, poor oral hygiene, and periodontal disease associated with helicobacter pylori infection?. J. Periodontol..

[30] Gao J., Li Y., Wang Q., Qi C., Zhu S. (2011). Correlation between distribution of helicobacter pylori in oral cavity and chronic stomach conditions. J. Huazhong Univ. Sci. Technol. Med. Sci..

[31] Al Asqah M., Al Hamoudi N., Anil S., Al Jebreen A., Al-Hamoudi W.K. (2009). Is the presence of helicobacter pylori in dental plaque of patients with chronic periodontitis a risk factor for gastric infection?. Can. J. Gastroenterol..

[32] Butt A.K., Khan A.A., Khan A.A., Izhar M., Alam A., Shah S.W., Shafqat F. (2002). Correlation of helicobacter pylori in dental plaque and gastric mucosa of dyspeptic patients. J. Pak. Med. Assoc..

[33] Zaric S., Bojic B., Jankovic L., Dapcevic B., Popovic B., Cakic S., Milasin J. (2009). Periodontal therapy improves gastric helicobacter pylori eradication. J. Dent. Res..

[34] Bouziane A., Ahid S., Abouqal R., Ennibi O. (2012). Effect of periodontal therapy on prevention of gastric helicobacter pylori recurrence: A systematic review and meta-analysis. J. Clin. Periodontol..

[35] Anand P.S., Kamath K.P., Anil S. (2014). Role of dental plaque, saliva and periodontal disease in helicobacter pylori infection. World J. Gastroenterol..

[36] Johnsen R., Forde O.H., Straume B., Burhol P.G. (1994). Aetiology of peptic ulcer: A prospective population study in Norway. J. Epidemiol. Community Health.

[37] Bytzer P., Howell S., Leemon M., Young L.J., Jones M.P., Talley N.J. (2001). Low socioeconomic class is a risk factor for upper and lower gastrointestinal symptoms: A population based study in 15000 Australian adults. Gut.

[38] Albandar J.M. (2002). Global risk factors and risk indicators for periodontal diseases. Periodontology.

[39] Croucher R., Marcenes W.S., Torres M.C., Hughes F., Sheiham A. (1997). The relationship between life-events and periodontitis. A case-control study. J. Clin. Periodontol..

[40] Klinge B., Norlund A. (2005). A socio-economic perspective on periodontal diseases: A systematic review. J. Clin. Periodontol..

